# A 16-year-old man with leptospirosis and atypical disseminated intravascular coagulation: a case report

**DOI:** 10.1186/s13256-023-04239-8

**Published:** 2023-11-27

**Authors:** Charlotte Conreur, Michelle Coureau, David Grimaldi, Olivier Simonet, Frédéric Vallot, Didier Ndjekembo Shango

**Affiliations:** 1 Department of Intensive Care, Centre Hospitalier de Wallonie Picarde, Rue des Sports, 51, 7500 Tournai, Belgium; 2 Department of Intensive Care, Erasme Hospital, Route de Lennik, 808, 1700 Brussels, Belgium

**Keywords:** Leptospirosis, Extracorporeal life support, Diffuse alveolar hemorrhage, Multiple organ failure, Case report

## Abstract

**Background:**

Leptospirosis is known for its pulmonary form characterized by intra-alveolar hemorrhage, exhibiting a high mortality rate. Management by venous–venous extracorporeal membrane oxygenation has been reported in a small number of cases.

**Case presentation:**

We report herein the case of a 16-year-old Caucasian male who was admitted with rapidly deteriorating respiratory and digestive complaints. He developed severe acute respiratory distress syndrome secondary to disseminated intravascular coagulation and intra-alveolar hemorrhage, requiring initiation of venous–venous extracorporeal membrane oxygenation. Initial infectious and immunological assessments were inconclusive, but repeat serology on the tenth day of admission confirmed a diagnosis of leptospirosis. The patient received multiple transfusions, and upon favorable response to treatment with corticosteroids and antibiotics, he was successfully weaned off venous–venous extracorporeal membrane oxygenation, which was discontinued after 12 days.

**Conclusion:**

Leptospirosis is a rare cause of severe acute respiratory failure following pulmonary hemorrhage. It is typically diagnosed by serology, with detectable IgM antibodies 5–7 days after the onset of symptoms. We report that early support with respiratory extracorporeal membrane oxygenation favors timely clearance of endobronchial clotting, parenchymal recovery, and prevention of ventilator-induced lung injury. Major hypofibrinogenemia, which did not seem to worsen during extracorporeal membrane oxygenation application, was managed by repeated transfusions. Further studies investigating the pathogenesis of this coagulopathy are required to further optimize the management of this rare and severe complication.

## Background

Leptospirosis is a zoonosis caused by the spirochete *Leptospira interrogans*. Human infection results from direct exposure to infected urine of carrier mammals, or from soil or water contaminated by wild mammals. Clinical manifestations vary from asymptomatic infection to severe illness with multiorgan dysfunction. Leptospirosis can cause sepsis and pulmonary hemorrhage associated with thrombocytopenia. Coagulopathy in severe leptospirosis remains poorly understood but is a major cause of death. We report a case of atypical disseminated intravascular coagulation associated with severe hypofibrinogenemia in an adolescent patient with leptospirosis.

## Case presentation

**Fig. 1:**
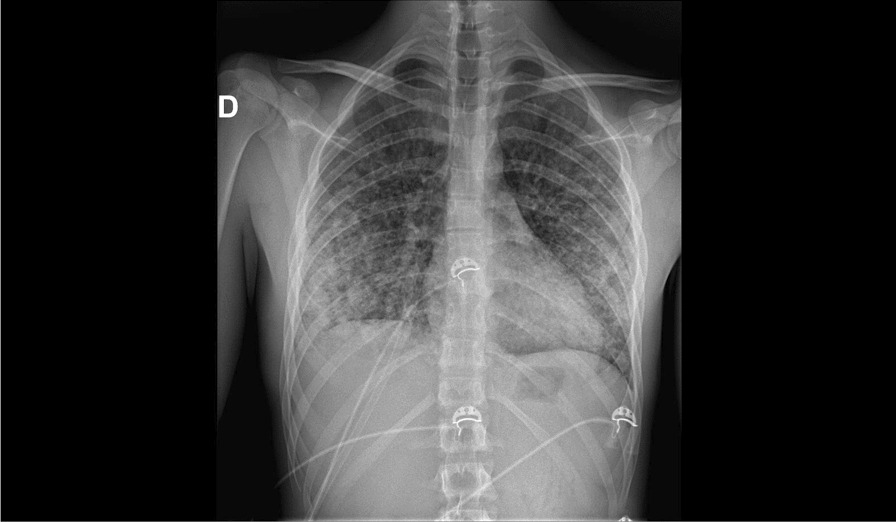
Chest radiography on arrival at the emergency room (H0): bilateral alveolar interstitial syndrome

**Fig. 2:**
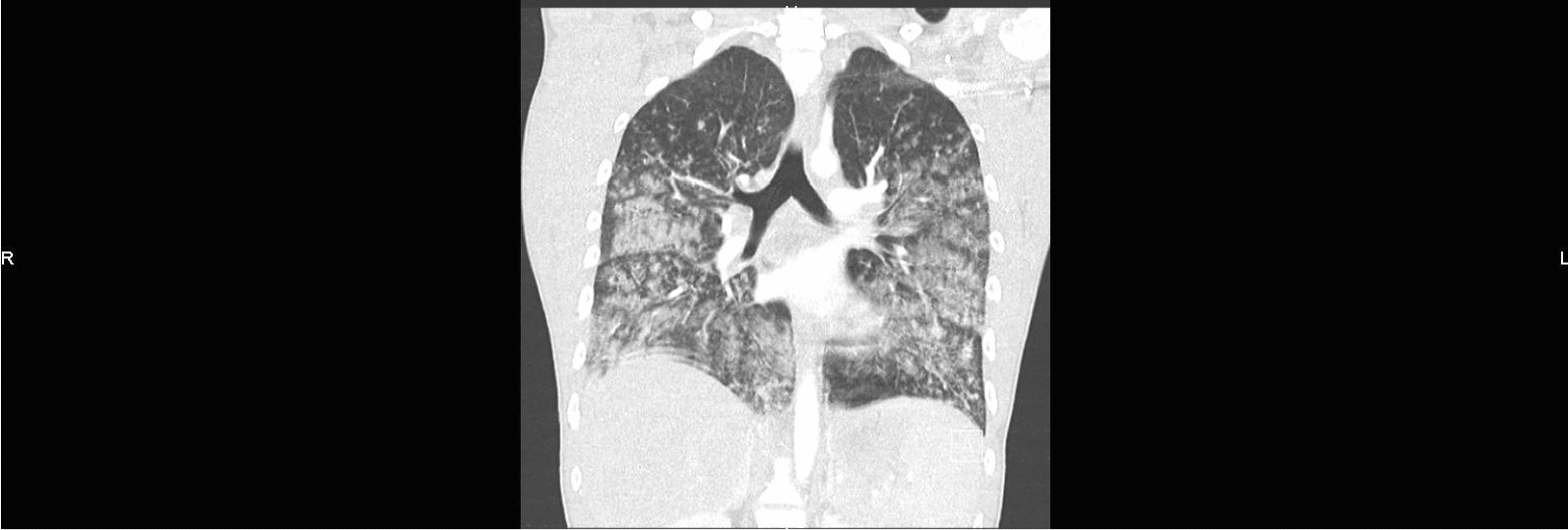
Chest scan on admission to intensive care (H + 6): diffuse alveolar syndrome with 80% lung damage

**Fig. 3:**
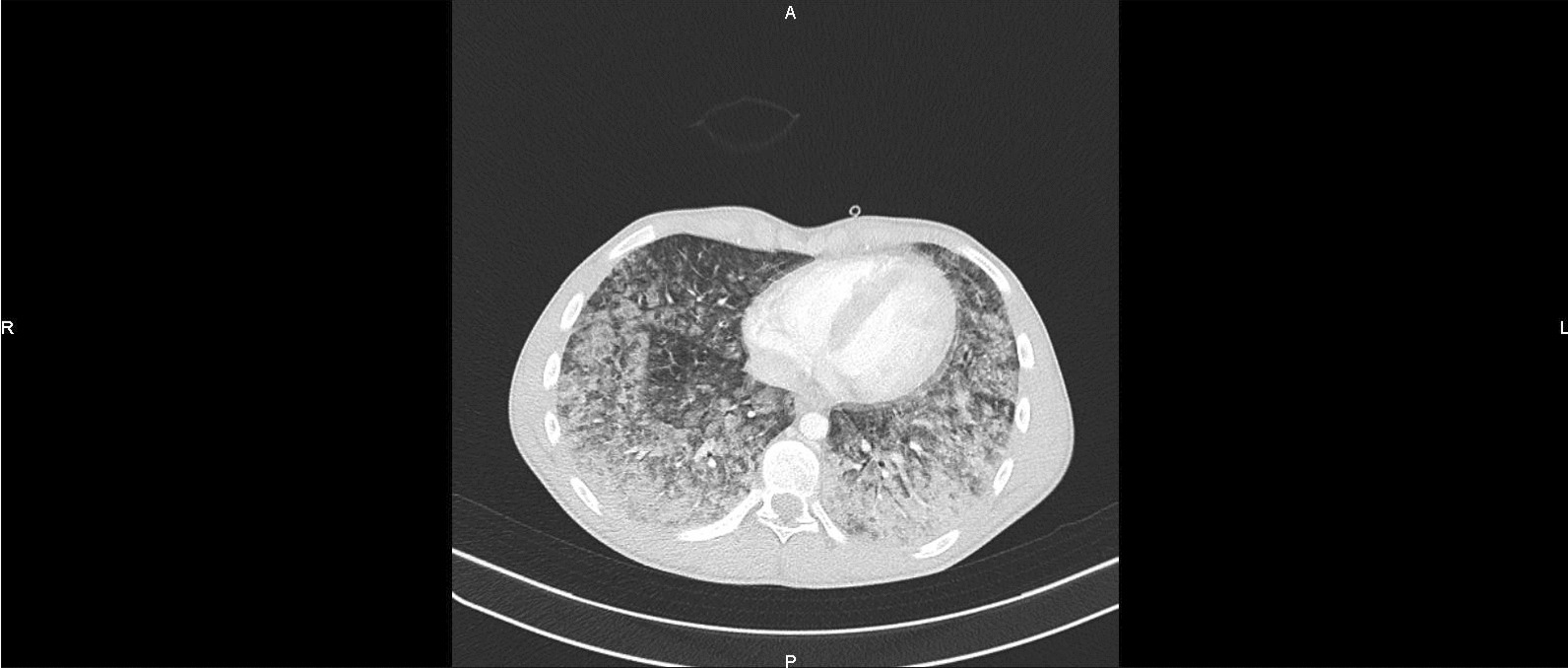
Chest scan on admission to intensive care (H + 6): diffuse alveolar syndrome with 80% lung damage

A 16-year-old Caucasian male presented to the emergency department with 3-day history of fatigue, fever, nausea, and diffuse myalgia. He also reported dyspnea and two episodes of hemoptysis, and on examination was found to be tachypneic, and tachycardic with pyrexia and transcutaneous oxygen saturation of 88% in room air, yet without signs of hemodynamic instability. There were bilateral crackles on pulmonary auscultation and diffuse pain on abdominal palpation without rebound or rigidity. The rest of the clinical examination was unremarkable. The chest radiography showed bilateral alveolar interstitial syndrome (Fig. 1). Laboratory studies on admission showed leukocytosis (8905/μl), thrombopenia (96,000/µl), and lymphopenia (398/μl), with mildly impaired coagulation parameters, including APTT of 27.3 s (normal range: 20–30 s), INR of 1.10 (normal range: < 1.10), PTT of 89% (normal range: 75–100%), and fibrinogen of 535 mg/dl (normal range: 200–400 mg/dl). A computed tomography (CT) scan of the chest showed bilateral consolidation with diffuse alveolar damage (Figs. 2 and 3). The patient was admitted, and antibiotic treatment was initiated with intravenous ceftriaxone.

**Fig. 4:**
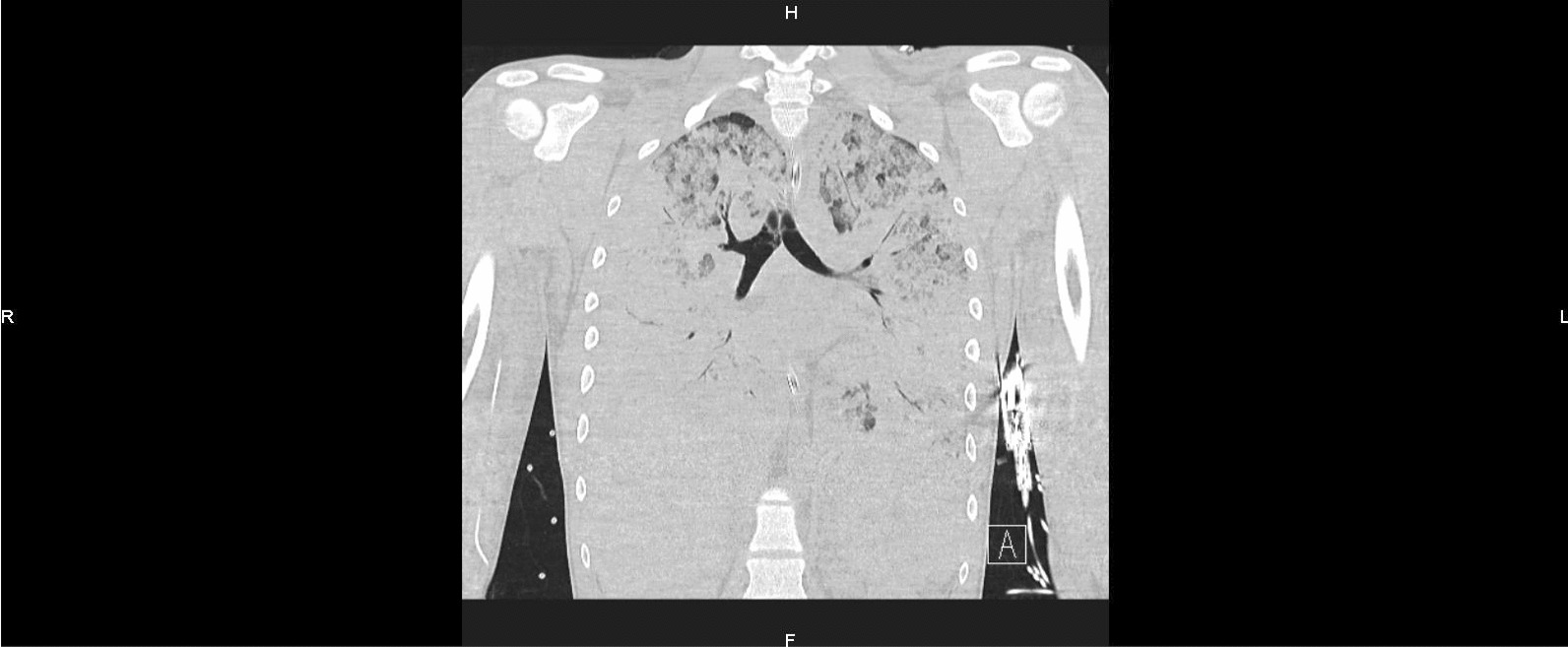
Chest scan 10 hours after admission to intensive care (H + 16): clear increase of pulmonary intraparenchymal lesions with complete involvement of both lungs

**Fig. 5:**
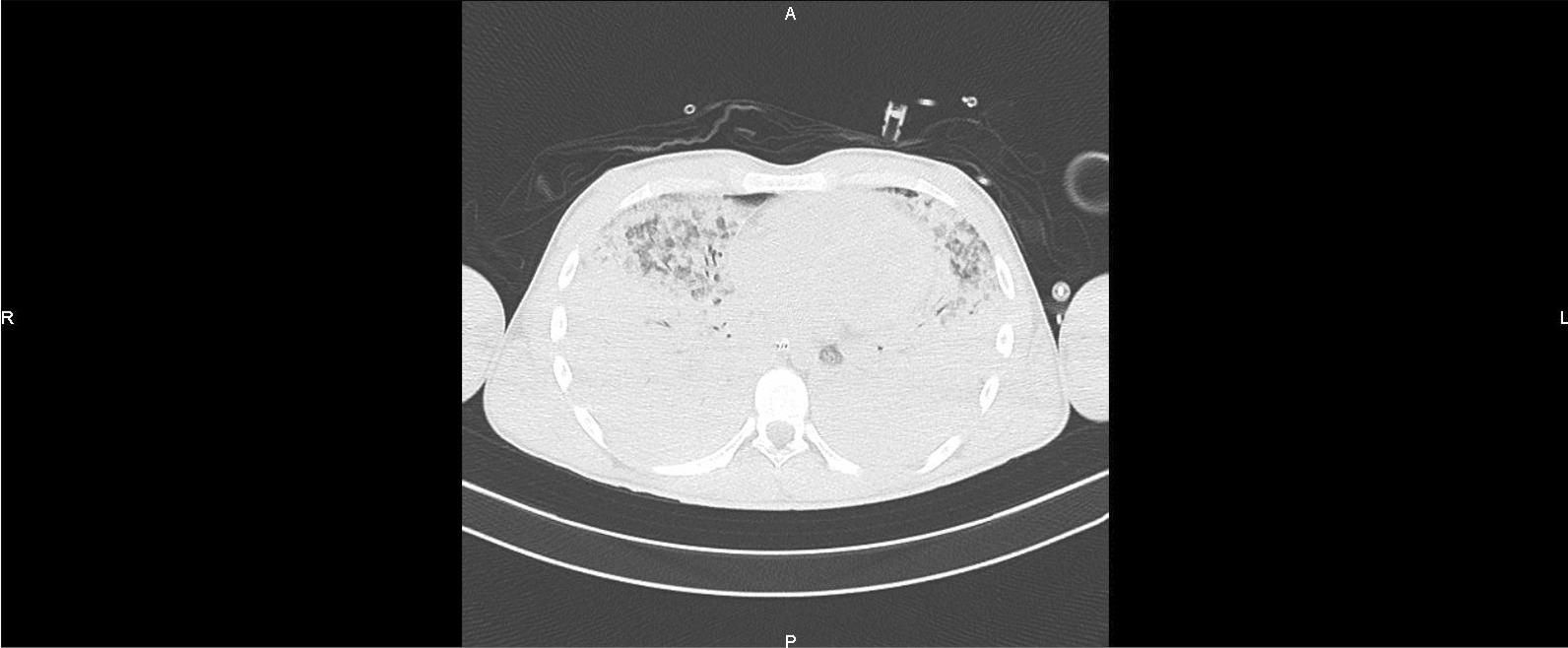
Chest scan 10 hours after admission to intensive care (H + 16): clear increase of pulmonary intraparenchymal lesions with complete involvement of both lungs

Within hours of hospital admission, the patient deteriorated and developed acute respiratory failure, requiring transfer to the intensive care unit for additional support, initially with supplemental high flow oxygen administration, but subsequently necessitating intubation and ventilation for persistent hypoxemia. Owing to refractory severe acute respiratory distress syndrome with PaO_2_/FiO_2_ ratio of 47, he was rapidly cannulated for venous–venous extracorporeal membrane oxygenation (vvECMO). Airway pressure release ventilation was used to ensure lung-protective ventilation, but examination by fibroscopy showed a pulmonary hemorrhage, requiring a massive transfusion with guidance by rotational thromboelastometry. A second chest scan showed a clear increase of pulmonary intraparenchymal lesions with complete involvement of both lungs (Figs. 4 and 5).

Owing to persistent cannulation site bleeding with recurrence of diffuse alveolar hemorrhage, aggravating the patient’s refractory severe acute respiratory distress syndrome, he was transferred to a university hospital for further management. He was also noted to have worsening coagulation dysfunction with fibrinogen at 153 mg/dl, d-dimer at 20563 ng/ml, INR of 1.19, APTT of 31.7 s, and platelet counts further decreasing to 25,000/μl. The patient developed jaundice with conjugated bilirubinemia of 6.7 mg/dl. There was no acute renal injury. Systemic anticoagulation to maintain circuit patency was stopped after 1 day. Overall, the persistent disseminated intravascular coagulation with severe hypofibrinogenemia (nadir at 79 mg/dl) was associated with rotational thromboelastometry results, justifying massive transfusions. Over a period of 7 days, the patient received 2 units of packed red blood cells, 2 units of platelets, 77 g of fibrinogen, 3 units of fresh frozen plasma, and 2800 UI of prothrombin–proconvertin–Stuart factor.

In-depth investigations were performed upon admission, including a comprehensive panel of microbial serologies and PCRs and vasculitis screens. All results came back negative. Serologies were repeated on day 10 of admission, and antibodies were detected against *Leptospira* with micro-agglutination assay at titer of 1:1600. Antibiotic treatment with intravenous ceftriaxone was continued, completing a course of 10 days. Despite the absence of anticoagulation, the ECMO transmembrane pressure remained stable. The patient showed clinical improvement with normalization of laboratory parameters, and was progressively weaned off ECMO. He was decannulated on day 12 and extubated on day 18, upon which he was transferred back to his local hospital and made a full recovery.

## Discussion and conclusion

Lung involvement in leptospirosis is common, and has been reported in 85% of patients in some case series [1]. Hematological features are also frequent, including thrombocytopenia and disseminated intravascular coagulapathy, associated with clinical bleeding, in particular pulmonary. For example, disseminated intravascular coagulation was found in 46% of cases in a Thai series [2]. The physiopathology underlying the coagulopathy in the context of leptospirosis is not well understood. According to recent publications, *Leptospira* and their toxins act on endothelial cell surface receptors, causing loss of vascular integrity, vascular leakage, and multiorgan failure [3]. Severe hypofibrinogenemia, as observed in our patient, has not yet been reported, but in a hemorrhagic situation, maintaining a fibrinogen level above 1 g/l [4] has been recommended.

Symptoms such as cough, dyspnea, and hemoptysis generally appear 4–6 days after infection, and can quickly progress to severe respiratory failure, which has been linked to lesional edema and intra-alveolar hemorrhage. Severe acute respiratory distress syndrome and bleeding disorders are responsible for a high mortality rate. The use of ECMO despite the hemorrhagic context has been reported in a number of cases, and nevertheless seems to be associated with a lower mortality rate [5]. ECMO can be a contributing factor to the coagulopathy, resulting in an acquired von Willebrand syndrome, but is not typically associated with disseminated intravascular coagulation. Moreover, heparin therapy is usually required during ECMO, but in view of persistent bleeding, it was not possible to follow this routine recommendation in our patient. We did not observe any prothrombotic complications despite the lack of anticoagulation.

The use of other treatments in addition to antimicrobial treatment, such as plasmapheresis and corticosteroid therapy, has been reported in severe leptospirosis, but no clear benefit has been established for these treatments compared with standard antibiotic therapy [6, 7]. Better outcomes are associated with early start of antibiotherapy, with the risk of developing severe disease being considerably higher if antibiotics are started later than 2 days after symptom onset [8, 9].

Diagnosis of leptospirosis is based on assays for bacteriological and serological detection, with serological assays more widely used. Bacteriological detection is possible by direct examination, culture, and PCR, but isolating the organism is challenging. Serological assays determine antibody titers against *Leptospira*, but their relevance depends on the time of infection. IgM antibodies appear from day 5 to day 7 after infection. The sensitivity and specificity of the enzyme-linked immunosorbent assay are 89% and 94%, respectively, while those of the microscopic agglutination test are 63% and 97%. A single titer of > 1:800 is sufficient to confirm an active or recent infection. A negative result does not rule out infection, and it is recommended to repeat the analysis 1–2 weeks later, especially in unexplained pulmonary hemorrhage even without suggestive history [10]. Antibiotic treatment for leptospirosis should not be delayed by the lack of a positive serology test for this potentially lethal disease.

In conclusion, we present a case of severe acute respiratory failure following pulmonary hemorrhage due to leptospirosis, demonstrating the importance of considering this rare differential in patients with unexplained pulmonary hemorrhage. Most cases of leptospirosis are diagnosed by serology, and IgM antibodies are detectable in the blood 5–7 days after the onset of symptoms. A negative serology must be repeated 1–2 weeks after disease onset, and antibiotic treatment should not be delayed. We recommend early consideration for respiratory support by extracorporeal membrane oxygenation to enable clearance of endobronchial clotting, parenchymal recovery, and prevention of ventilator-induced lung injury. The pathogenesis of the associated coagulopathy remains poorly understood, but we show that it is possible to manage the major hypofibrinogenemia by transfusion and that the coagulopathy was not seemingly exacerbated by ECMO treatment in our patient.

## Data Availability

Not applicable.

## Abbreviations

vvECMO: Venous–venous extracorporeal membrane oxygenation
ARDS: Severe acute respiratory distress syndrome
DIC: Disseminated intravascular coagulation
PCR: Polymerase chain reaction
IgM: Immunoglobulin M
APTT: Activated partial thromboplastin time
INR: International normalized ratio
PTT: Prothrombin time
PaO2: Partial pressure of oxygen
FiO2: Fraction of inspired oxygen
APRV: Airway pressure release ventilation
ROTEM: Rotative thromboelastometry
MAT: Microscopic agglutination test
ELISA: Enzyme-linked immunosorbent assay
